# Supplementary material for: Adaptive dynamics of extrachromosomal circular DNA in rice under nutrient stress
Source: Nat Commun. 2025 May 4;16:4150. doi: 10.1038/s41467-025-59572-x (PMC12050283; doi:10.1038/s41467-025-59572-x)
Supplement: Supplementary file 1 — Supplementary Figs. [file 41467_2025_59572_MOESM1_ESM.pdf]

Supplementary Figures

Dynamics of eccDNAs in rice and their implication for adaptation to nutritional stress

Hanfang Ni[1,2#], Lenin Yong-Villalobos[3#], Mian Gu[1,2], Damar Lizbeth López-Arredondo[3], Min Chen[1,2], Liyan Geng[1,2], Guohua Xu[1,2]* and Luis Rafael Herrera-Estrella[1,4]*

[1] National Key Laboratory of Crop Genetics & Germplasm Enhancement and Utilization, Nanjing Agricultural University, Nanjing 210095, China.

[2] MOA Key Laboratory of Plant Nutrition and Fertilization in Lower-Middle Reaches of the Yangtze River, Nanjing 210095, China.

[3] Department of Plant and Soil Science, Institute of Genomics for Crop Abiotic Stress Tolerance (IGCAST), Texas Tech University, Lubbock, TX 79409, USA.

[4] Unidad de Genómica Avanzada/Langebio, Centro de Investigación y de Estudios Avanzados del Instituto Politécnico Nacional, Irapuato, Gto. 36821, Mexico.

* Correspondence: Guohua Xu (ghxu@njau.edu.cn); Luis Rafael Herrera-Estrella (luis.herrera-estrella@ttu.edu)

[#] These authors equally contributed to this work: Hanfang Ni, Lenin Yong-Villalobos

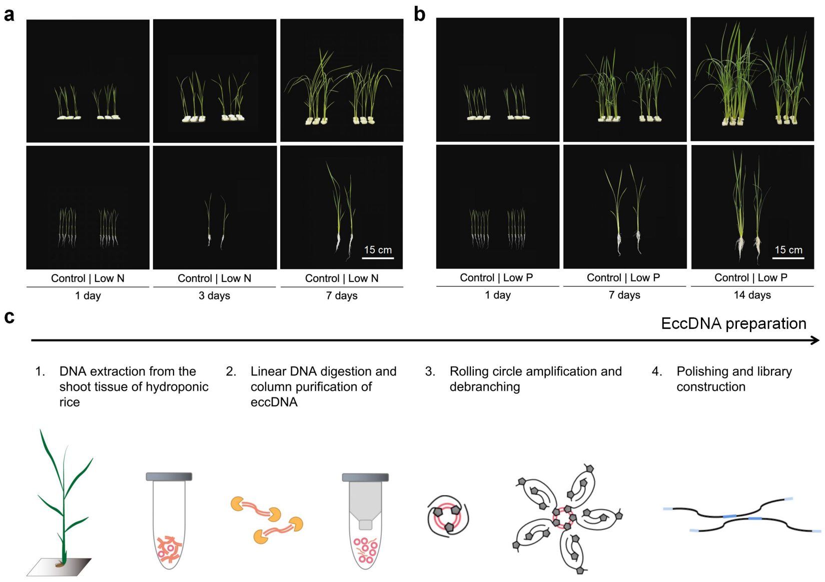

Supplementary Figure 1. Rice hydroponic treatments and eccDNA preparation processes.

a, Growth phenotype of Nipponbare rice plants from short-term (3 days) and long-term (7 days) in control (N concentration: 1.25 mM) and low nitrogen (N concentration: 0.25 mM) conditions. **b,** Growth phenotype of Nipponbare rice plants from short-term (7 days) and long-term (14 days) in control (P concentration: 0.1 mM) and low phosphorous (P concentration: 5 μM) conditions. **c,** Schematic diagrams of eccDNA isolation, purification, amplification and processing for Oxford Nanopore libraries preparation. Short double curves in orange: double-strand linear DNAs; double circles in red: double-strand circular DNAs; sectors in orange: ATP-dependent PlasmidSafe DNase; pentagons in grey: phi29 DNA Polymerase; single-strand curves in black: hyper-branched DNAs after random Rolling Circle Amplification and debranched DNAs after polishing; short lines in blue: showing gap-filling; short lines in light blue: showing polishing.

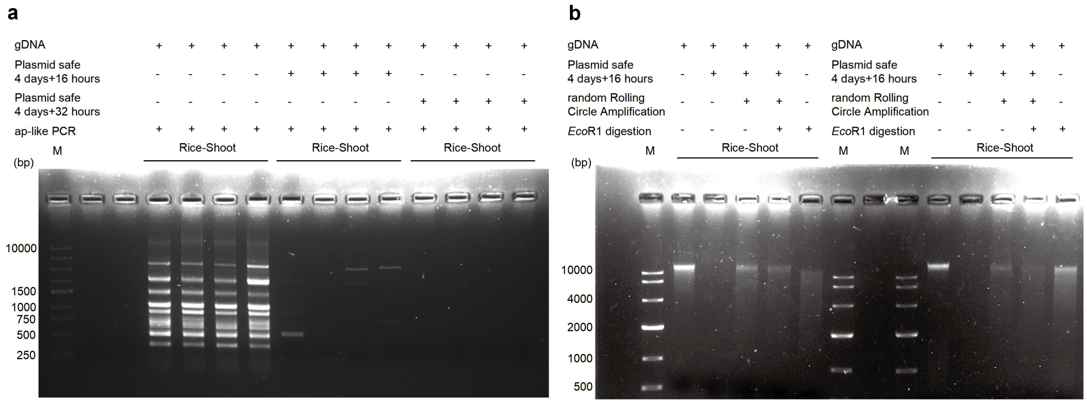

Supplementary Figure 2. Validation of linear DNA digestion and random Rolling Circle Amplification.

a, Arbitrarily primed-like PCR (ap-like PCR) validation of PlasmidSafe enzyme digestion. Both genomic DNAs (gDNA) and pure eccDNAs after ATP-dependent PlasmidSafe DNase reaction (Plasmid safe) were used as the templates for ap-like PCR to confirm the successful digestion on linear DNAs. The experiment was conducted twice. Besides the extraction of gDNA and linear DNA digestion mentioned previously, a pair of non-specific primers on *OsActin-1* (LOC4338914) were designed as: forward primer: 5'-TGGCTGACGAGGATATTCA-3'; reverse primer, 5'-TGCAGTATTGGACGCTAACA-3' for ap-like PCR. The PCR was performed following manufacturer's instructions of 2× Taq Mastermix (CWBIO Co., Cat. No. CW0682M). 50°C and 30 seconds were used for annealing, followed by 72°C for 2 min for extension; 40 cycles were run. PCR products were analyzed through agarose gel electrophoresis. M: DNA Marker; bp: base pair. **b,** *Eco*R1 digestion validation of random Rolling Circle Amplification (rRCA). Both gDNA and the products from rRCA were used as the templates for *Eco*R1 digestion validation. The experiment was conducted twice. The digestion followed the manufacturer's instructions for *Eco*RI-HF® (New England Biolabs, Cat. No. R3195S) and incubated at 37°C for 12 h. Digestion products with gDNA, pure eccDNAs after ATP-dependent PlasmidSafe DNase reaction, and rRCA products were analyzed through agarose gel electrophoresis. M: DNA Marker; bp: base pair.

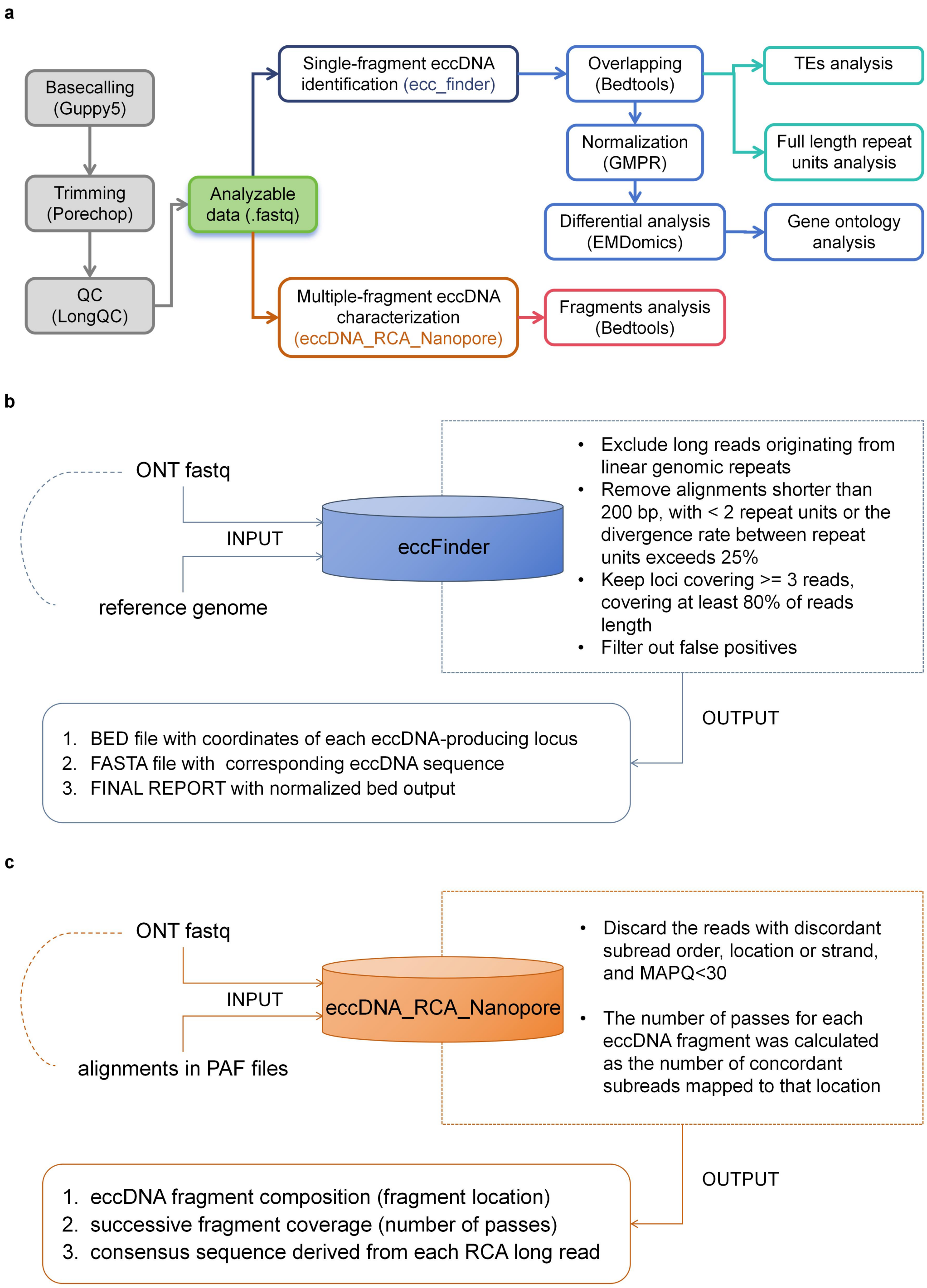

Supplementary Figure 3. Bioinformatic identification of rice eccDNAs.
a, Workflow for identification and differential analysis of eccDNAs (QC: quality control). **b,** Workflow for eccDNA identification through ecc_finder pipeline (ONT fastq: sequencing data in fastq format generated from Oxford Nanopore Technology). **c,** Workflow for eccDNA identification through eccDNA_RCA_Nanopore pipeline.

a

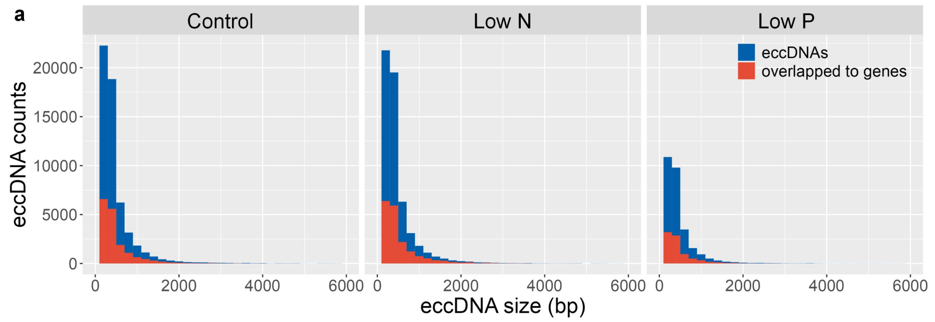

b

	median (bp)	min (bp)	max (bp)	average (bp)	mode (bp)
all Control	338	200	37214	504	215
all Low N	342	200	37072	504	217
all Low P	344	200	37138	516	217

Supplementary Figure 4. Basic analysis of eccDNAs under different nutritional treatments.

a, Size distribution of eccDNAs (Blue bars) and eccDNAs that overlap with genes (Red bars) separately identified in control samples (Control), low nitrogen treatments (Low N) and low phosphorous treatments (Low P). Only the most abundant eccDNAs ranging from 200 bp to 6 kb are shown. **b,** Basic statistics analysis on the size of eccDNAs identified in Control, Low N, and Low P. bp: base pair.

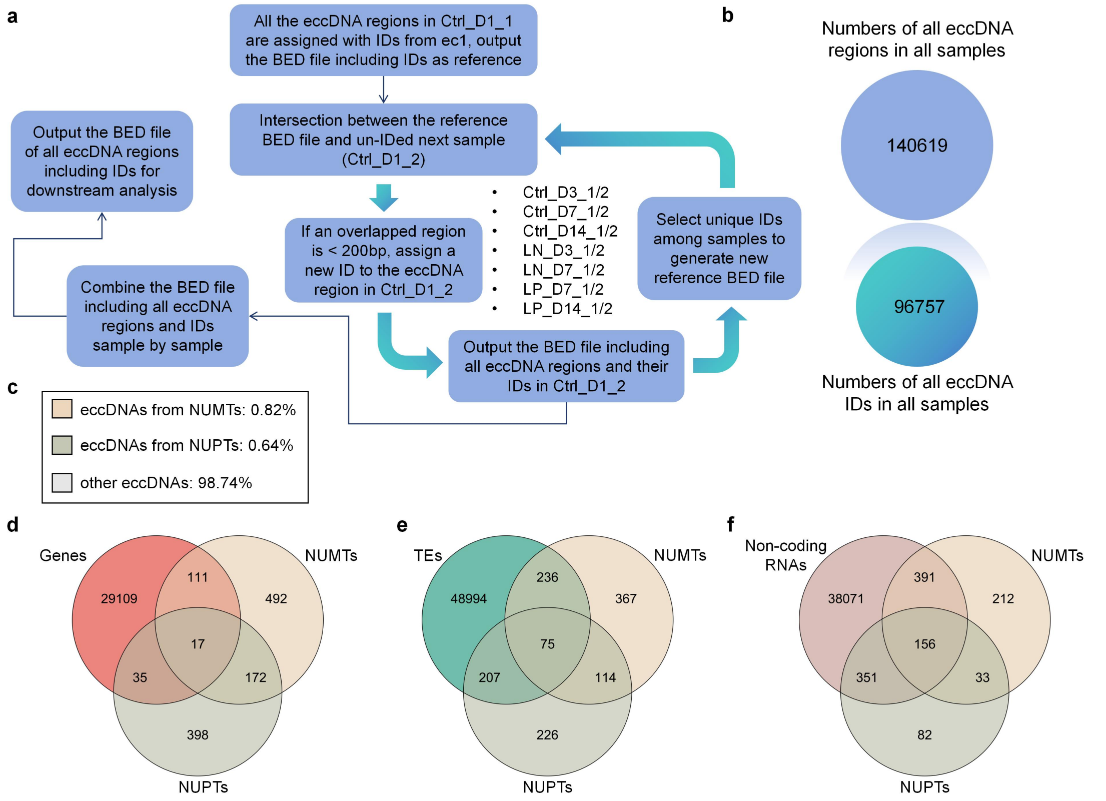

Supplementary Figure 5. ID assignment for eccDNA regions and analysis of eccDNAs from the nuclear mitochondrial DNA (NUMTs) and nuclear plastid DNA (NUPTs).

a, Workflow for ID assignment for eccDNA regions across all samples. **b,** Counts of total eccDNA regions and individual eccDNA IDs. **c,** Proportion of eccDNAs origin from NUMTs and NUPTs in all samples. **d,** Venn diagram showing all *ecGenes* (Genes in red), overall eccDNAs origin from NUMTs (NUMTs in cream yellow), and overall eccDNAs origin from NUPTs (NUMTs in cream green). **e,** Venn diagram showing all *ecTEs* (TEs in green), overall eccDNAs origin from NUMTs (NUMTs in cream yellow), and overall eccDNAs origin from NUPTs (NUMTs in cream green). **f,** Venn diagram showing all *ecNon-codingRNAs* (Non-coding RNAs in pink), overall eccDNAs origin from NUMTs (NUMTs in cream yellow), and overall eccDNAs origin from NUPTs (NUMTs in cream green).

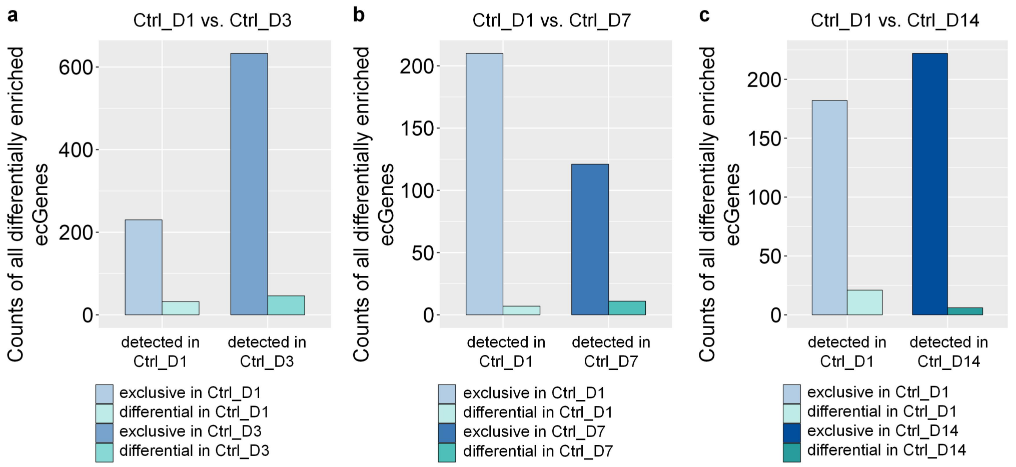

Supplementary Figure 6. Counts of exclusive *ecGenes* and differential *ecGenes* during rice optimal growth.

a, Count distribution of both exclusive and differential *ecGenes* identified in 1-day control samples (*Ctrl_D1*) and 3-day control samples (*Ctrl_D3*). **b,** Count distribution of both exclusive and differential *ecGenes* identified in *Ctrl_D1* and 7-day control samples (*Ctrl_D7*). **c,** Count distribution of both exclusive and differential *ecGenes* identified in *Ctrl_D1* and 14-day control samples (*Ctrl_D14*).

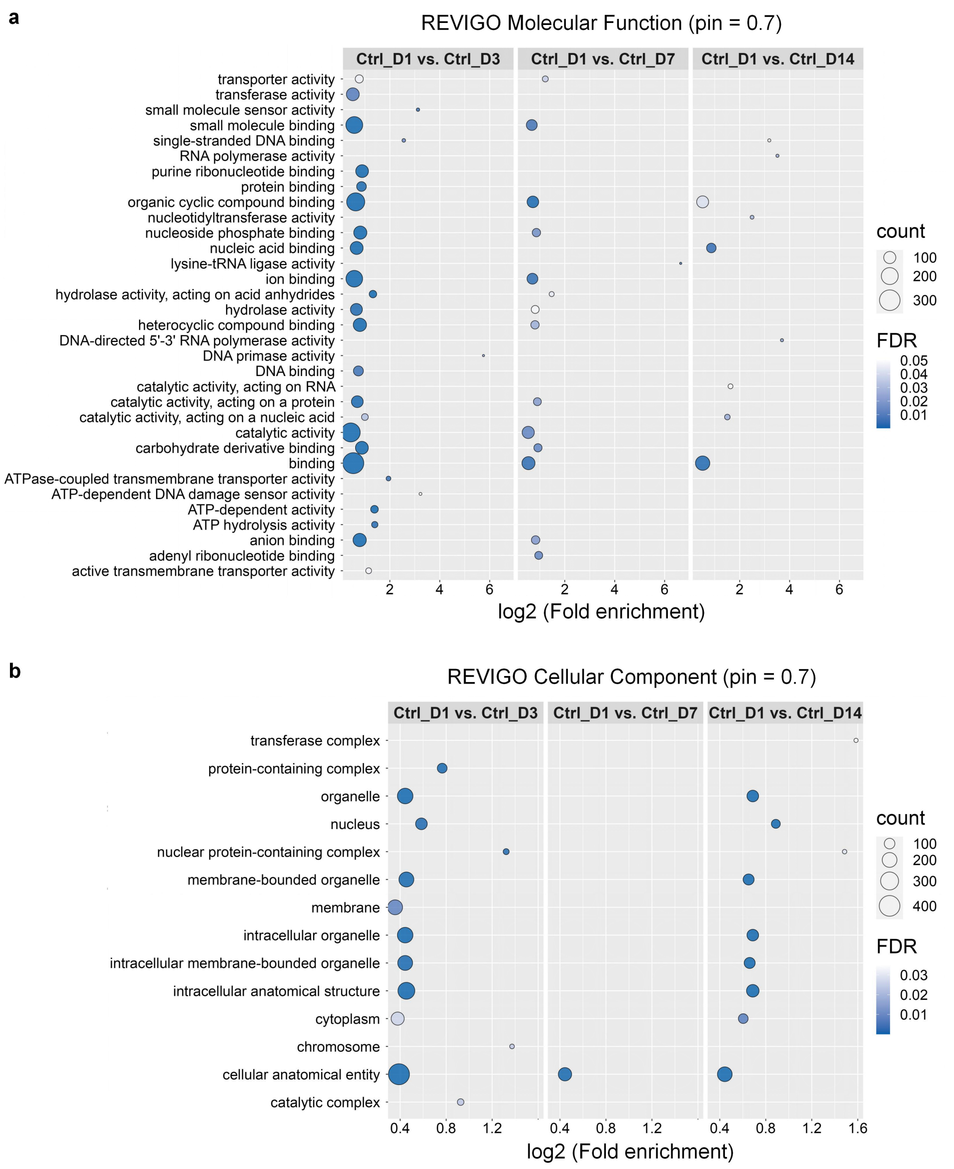

Supplementary Figure 7. Molecular function and cellular component GO domains summarized by REVIGO on exclusive *ecGenes* during rice optimal growth.

a, Dot-plot illustrating the significantly enriched GO molecular function categories summarized by REVIGO for the exclusive *ecGenes* among 1-day control samples *vs.* 3-day control samples (*Ctrl_D1 vs. Ctrl_D3*), 1-day control samples *vs.* 7-day control samples (*Ctrl_D1 vs. Ctrl_D7*) and 1-day control samples vs. 14-day control samples (*Ctrl_D1 vs. Ctrl_D14*). **b,** Dot-plot illustrating the significantly enriched GO cellular component categories summarized by REVIGO for the exclusive *ecGenes* among *Ctrl_D1 vs. Ctrl_D3*, *Ctrl_D1 vs. Ctrl_D7* and *Ctrl_D1 vs. Ctrl_D14*.

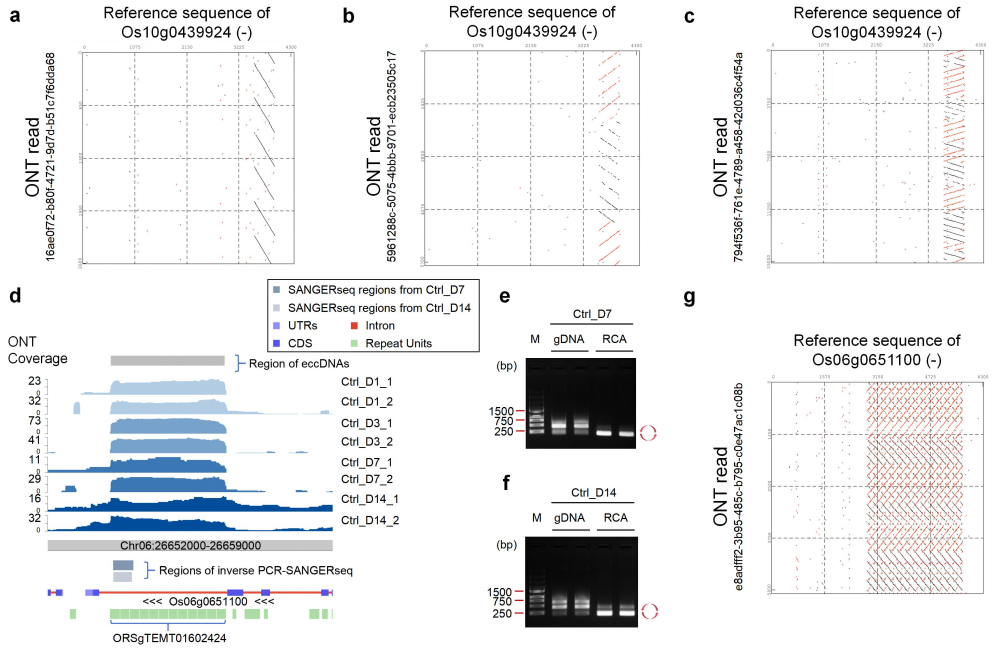

Supplementary Figure 8. Sequence analysis and validation on *ecGenes* during rice optimal growth.
a, Dot-plot showing correlation between individual sequence from Nanopore sequencing (ONT) read - 16ae0f72-b80f-4721-9d7d-b51c7f6dda68 and reference sequence of Os10g0439924 in one replicate of 14-day control samples (*Ctrl_D14_2*) (Black dots: Seq2PlusStrand; Red dots: Seq2MinusStrand). **b,** Dot-plot showing correlation between individual sequence from ONT read - 5961288c-5075-4bbb-9701-ecb23505c17 and reference sequence of Os10g0439924 in one replicate of 14-day control samples (*Ctrl_D14_2*) (Black dots: Seq2PlusStrand; Red dots: Seq2MinusStrand). **c,** Dot-plot showing correlation between individual sequence from ONT read -794f536f-761e-4789-a458-42d036c4f54a and reference sequence of Os10g0439924 in one replicate of 14-day control samples (*Ctrl_D14_2*) (Black dots: Seq2PlusStrand; Red dots: Seq2MinusStrand). **d,** Karyoplots of the Chr06:26652000-26659000 locus showing (from top to bottom) regions of eccDNAs, ONT coverage in two replicates of all control samples (*Ctrl_D1_1* and *Ctrl_D1_2*, *Ctrl_D3_1* and *Ctrl_D3_2*, *Ctrl_D7_1* and *Ctrl_D7_2*, *Ctrl_D14_1* and *Ctrl_D14_2*), regions of SANGER sequencing (SANGERseq) fragments from inverse PCR validation in 7-day and 14-day control samples, the transcript structure of Os06g0651100, and regions of repeat unit including a set of *ORSgTEMT01602424* elements. **e,** Gel electrophoresis of inverse PCR products from the *ecGene* derived from the Os06g0651100 locus in 7-day control samples (*Ctrl_D7*). The experiment was conducted twice. M: DNA Marker; bp: base pair; gDNA: template genomic DNA from *Ctrl_D7*; RCA: template rRCA products from *Ctrl_D7*; forward primer: 5'-CATGTTTGACCGTTCATCTTATTC-3'; reverse primer: 5'-CATTATATCCAGAATACTCTCTCTC-3'; circle in red: amplified products of *ecGene*. **f,** Gel electrophoresis of inverse PCR products from the *ecGene* derived from the Os06g0651100 locus in 14-day control samples (*Ctrl_D14*). The experiment was conducted twice. M: DNA Marker; bp: base pair; gDNA: template genomic DNA from *Ctrl_D14*; rRCA: template rRCA products from *Ctrl_D14*; forward primer: 5'-CATGTTTGACCGTTCATCTTATTC-3'; reverse primer: 5'-CATTATATCCAGAATACTCTCTCTC-3'; circles in red: symbol used for pointing out amplified products of *ecGene*. **g,** Dot-plot showing correlation between individual sequence from one ONT read and reference sequence of Os06g0651100 in one replicate of 14-day control samples (*Ctrl_D14_2*) (Black dots: Seq2PlusStrand; Red dots: Seq2MinusStrand).

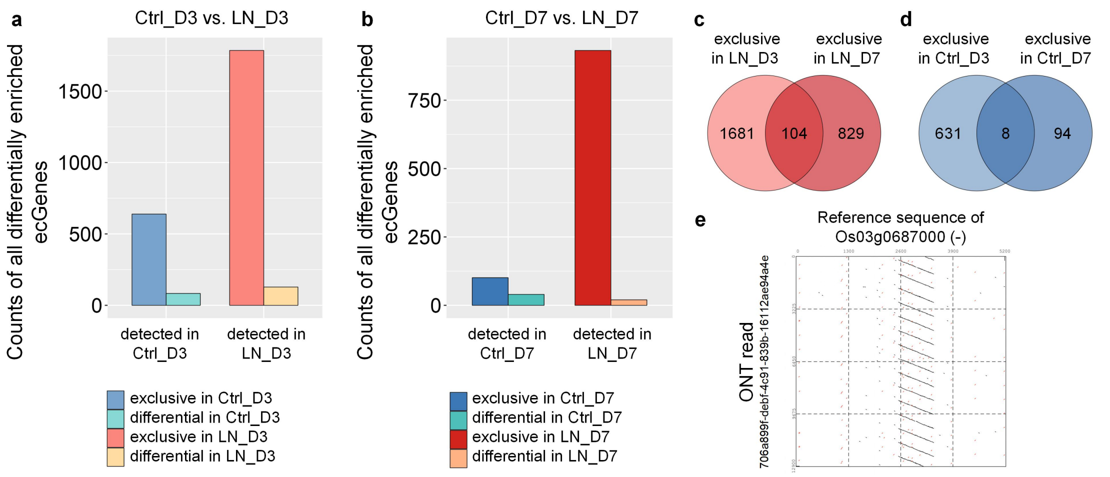

Supplementary Figure 9. Counts of exclusive *ecGenes* and differential *ecGenes* together with exclusive *ecGene* analysis under low nitrogen (LN) treatments.
a, Count distribution of both exclusive and differential *ecGenes* identified in 3-day control samples (*Ctrl_D3*) and 3-day LN treatments (*LN_D3*). **b,** Count distribution of both exclusive and differential *ecGenes* identified in 7-day control samples (*Ctrl_D7*) and 7-day LN treatments (*LN_D7*). **c,** Venn diagram of exclusive *ecGenes* in *LN_D3* (in pink) and *LN_D7* (in red). **d,** Venn diagram of exclusive *ecGenes* in *Ctrl_D3* (in light blue) and *Ctrl_D7* (in blue). **e,** Dot plot showing correlation between individual sequence from one Nanopore sequencing (ONT) read in one replicate of 7-day LN treatment (*LN_D7_2*) and reference sequence of Os03g0687000 (Black dots: Seq2PlusStrand; Red dots: Seq2MinusStrand).

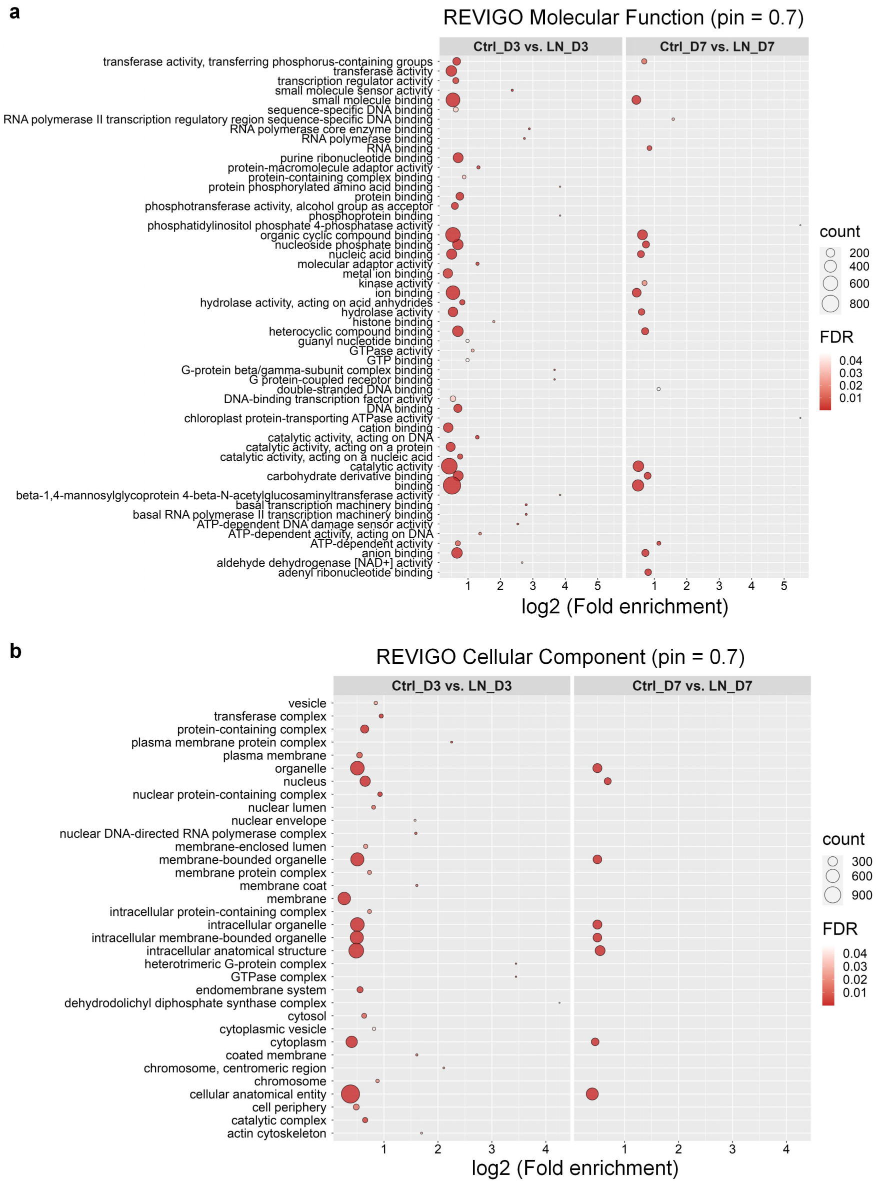

Supplementary Figure 10. Molecular function and cellular component GO domains summarized by REVIGO on exclusive *ecGenes* under low nitrogen (LN) treatments. **a,** Dot-plot illustrating the significantly enriched GO molecular function categories summarized by REVIGO for the exclusive *ecGenes* from both 3-day control samples *vs*. 3-day LN treatments (*Ctrl_D3 vs*. *LN_D3*) and 7-day control samples *vs*. 7-day LN treatments (*Ctrl_D7 vs*. *LN_D7*). **b,** Dot-plot illustrating the significantly enriched GO cellular component categories summarized by REVIGO for the exclusive *ecGenes* from both *Ctrl_D3 vs*. *LN_D3* and *Ctrl_D7 vs*. *LN_D7*.

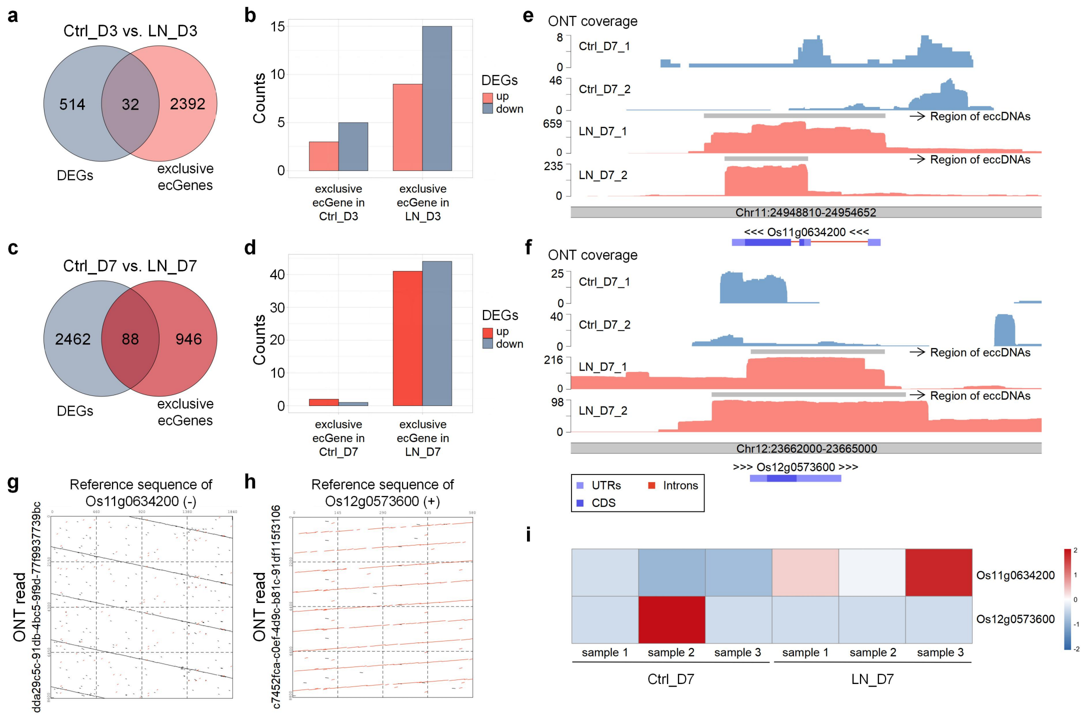

Supplementary Figure 11. Association analysis between differentially expressed genes (DEGs) and exclusive *ecGenes* together with the full-length *ecGene* analysis under low nitrogen (LN) treatments.

a, Venn diagram of DEGs (in gray blue) and exclusive *ecGenes* (in pink) identified in 3-day control samples vs. 3-day LN treatments (*Ctrl_D3* vs. *LN_D3*). **b,** Count distribution of exclusive *ecGenes* in *Ctrl_D3* or *LN_D3* that also classified as up- or down- regulated DEGs. **c,** Venn diagram of DEGs (in gray blue) and exclusive *ecGenes* (in red) identified in 7-day control samples vs. 7-day LN treatments (*Ctrl_D7* vs. *LN_D7*). **d,** Count distribution of exclusive *ecGenes* in *Ctrl_D7* or *LN_D7* that also classified as up- or down-regulated DEGs. **e,** Karyoplots of the Chr11:24948810-24954652 locus showing (from top to bottom) Nanopore sequencing (ONT) coverage in two replicates of 7-day control samples (*Ctrl_D7_1* and *Ctrl_D7_2*), regions of eccDNAs and ONT coverage in two replicates of 7-day LN treatments (*LN_D7_1* and *LN_D7_2*), and the transcript structure of Os11g0634200. **f,** Karyoplots of the Chr12:23662000-23665000 locus showing (from top to bottom) ONT coverage in two replicates of 7-day control samples (*Ctrl_D7_1* and *Ctrl_D7_2*), regions of eccDNAs and ONT coverage in two replicates of 7-day LN treatments (*LN_D7_1* and *LN_D7_2*), and the transcript structure of Os12g0573600. **g,** Dot plot showing correlation between individual sequence from one ONT read in one replicate of 7-day LN treatments (*LN_D7_1*) and reference sequence of Os11g0634200 (Black dots: Seq2PlusStrand; Red dots: Seq2MinusStrand). **h,** Dot-plot showing correlation between individual sequence from one ONT read in one replicate of 7-day LN treatments (*LN_D7_1*) and reference sequence of Os12g0573600 (Black dots: Seq2PlusStrand; Red dots: Seq2MinusStrand). **i,** Heat map showing the normalized expression level as log2(normalized counts +1) of Os11g0634200 and Os12g0573600 as DEGs by each sample in RNAseq.

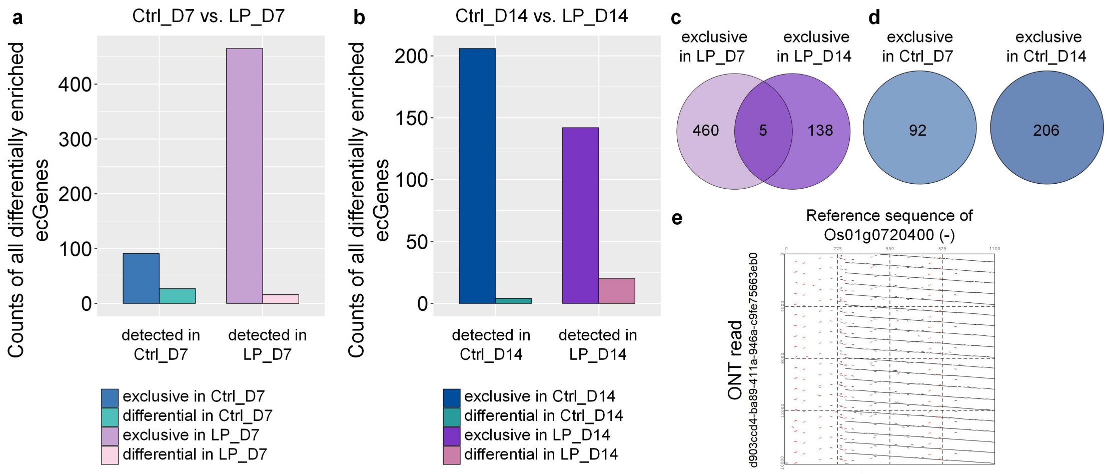

Supplementary Figure 12. Counts of exclusive *ecGenes* and differential *ecGenes* together with exclusive *ecGene* analysis under low phosphorus (LP) treatments.

a, Count distribution of both exclusive and differential *ecGenes* identified in 7-day control samples (*Ctrl_D7*) and 7-day LP treatments (*LP_D7*). **b,** Count distribution of both exclusive and differential identified in 14-day control samples (*Ctrl_D14*) and 14-day LP treatments (*LP_D14*). **c,** Venn diagram of exclusive *ecGenes* in *LP_D7* (in light purple) and *LP_D14* (in purple). **d,** Venn diagram of exclusive *ecGenes* in *Ctrl_D7* (in light blue) and *Ctrl_D14* (in blue). **e,** Dot plot showing correlation between individual sequence from one Nanopore sequencing (ONT) read in one replicate of 14-day LP treatments (*LP_D14_1*) and reference sequence of Os01g0720400 (Black dots: Seq2PlusStrand; Red dots: Seq2MinusStrand).

a

b

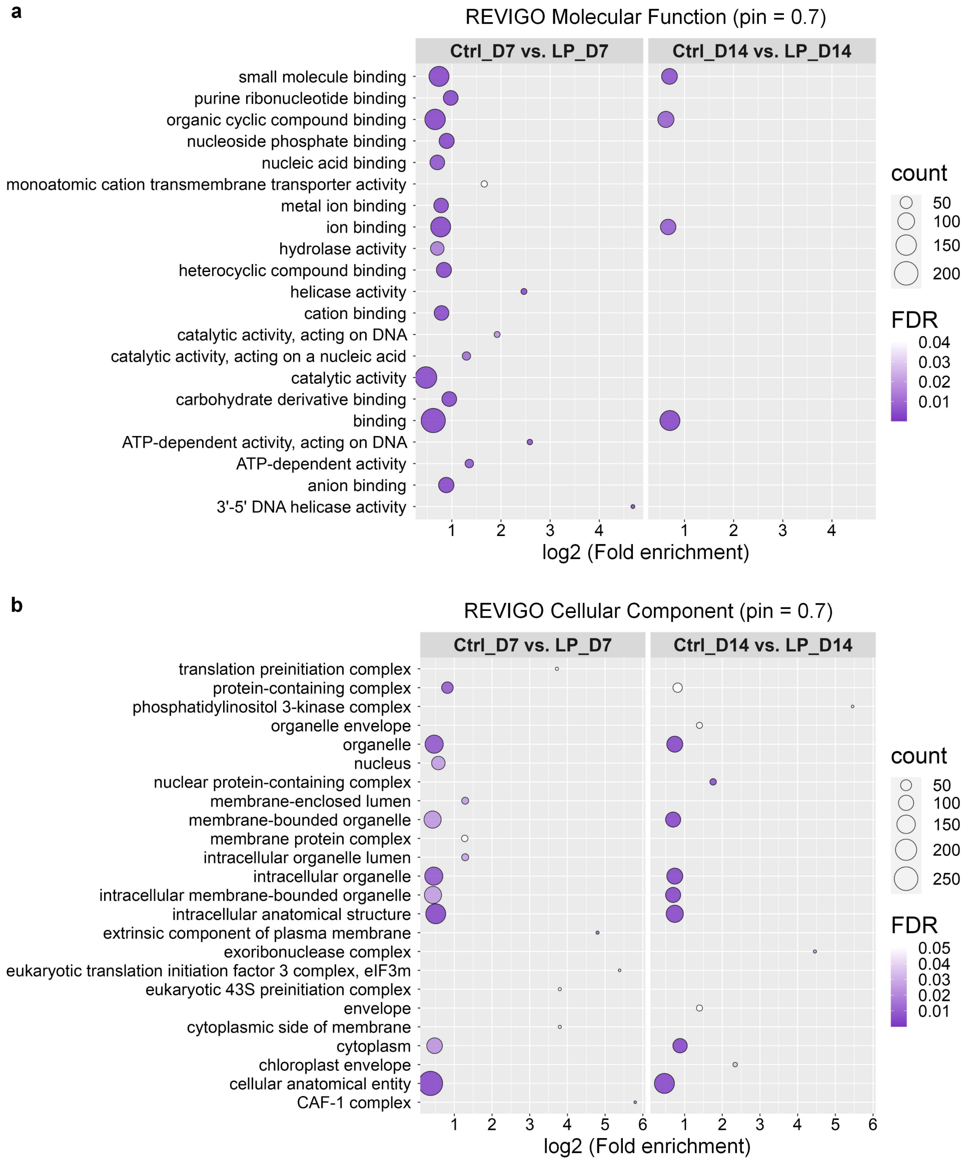

Supplementary Figure 13. Molecular function and cellular component GO domains summarized by REVIGO on exclusive *ecGenes* under low phosphorus (LP) treatments.

a, Dot-plot illustrating the significantly enriched GO molecular function categories summarized by REVIGO for the exclusive *ecGenes* from 7-day control samples vs. 7-day LP treatments (*Ctrl_D7* vs. *LP_D7*) and 14-day control samples vs. 14-day LP treatments (*Ctrl_D14* vs. *LP_D14*). **b,** Dot-plot illustrating the significantly enriched GO cellular component categories summarized by REVIGO for the exclusive *ecGenes* from *Ctrl_D7* vs. *LP_D7* and *Ctrl_D14* vs. *LP_D14*.

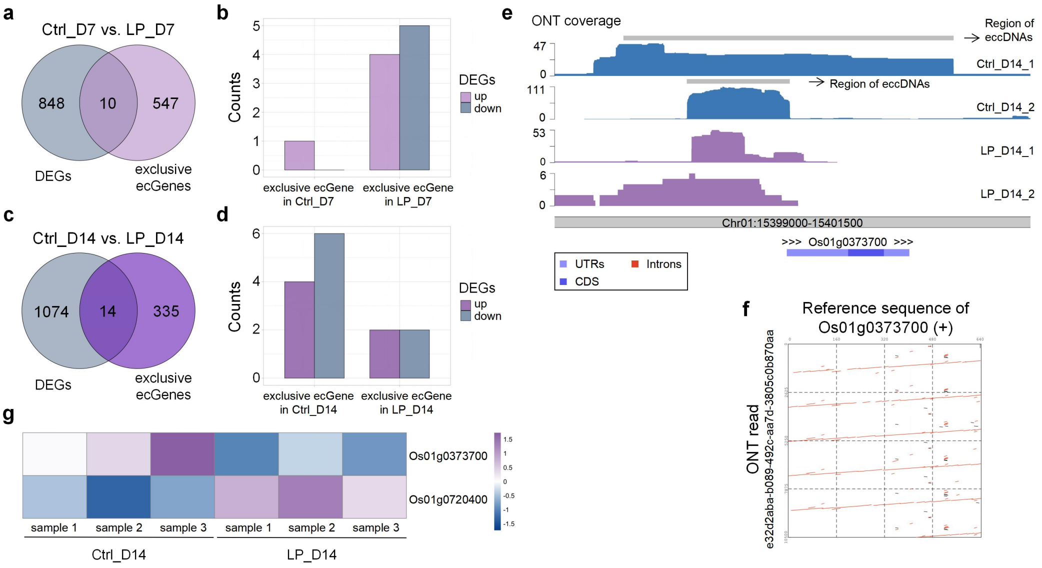

Supplementary Figure 14. Association analysis between differentially expressed genes (DEGs) and exclusive *ecGenes* together with the full-length *ecGene* analysis under low phosphorus (LP) treatments.

a, Venn diagram of DEGs (in gray blue) and exclusive *ecGenes* (in light purple) identified in 7-day control samples vs. 7-day LP treatments (*Ctrl_D7* vs. *LP_D7*). **b,** Count distribution of exclusive *ecGenes* in *Ctrl_D7* or *LP_D7* that also classified as up- or down-regulated DEGs. **c,** Venn diagram of DEGs (in gray blue) and exclusive *ecGenes* (in purple) identified in 14-day control samples vs. 14-day LP treatments (*Ctrl_D14* vs. *LP_D14*). **d,** Count distribution of exclusive *ecGenes* in *Ctrl_D14* or *LP_D14* that also classified as up- or down- regulated DEGs. **e,** Karyoplots of the Chr01:15399000-15401500 locus showing (from top to bottom) regions of eccDNAs and Nanopore sequencing (ONT) coverage in two replicates of 14-day control samples (*Ctrl_D14_1* and *Ctrl_D14_2*), ONT coverage in two replicates of 14-day LP treatments (*LP_D14_1* and *LP_D14_2*), and the transcript structure of Os01g0373700. **f,** Dot plot showing correlation between individual sequence from one ONT read in one replicate of 14-day control samples (*Ctrl_D14_1*) and reference sequence of Os01g0373700 (Black dots: Seq2PlusStrand; Red dots: Seq2MinusStrand). **g,** Heat map showing the normalized expression level as log2(normalized counts +1) of Os01g0373700 and Os01g0720400 as DEGs by each sample in RNAseq.

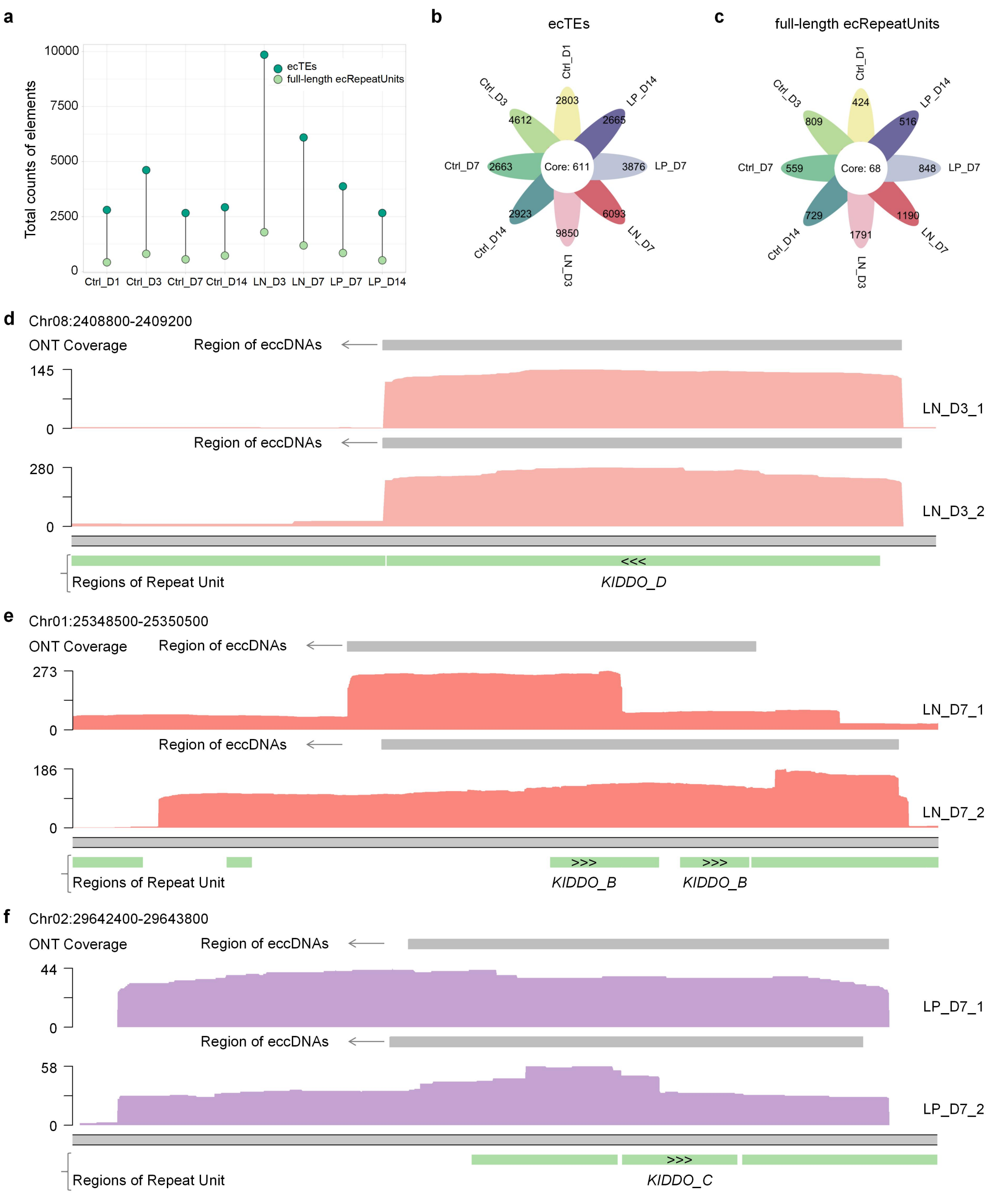

Supplementary Figure 15. Count distribution of *ecTEs* and *full-length ecRepeatUnits* in rice and analysis of *Kiddo* in *full-length ecRepeatUnits*.

a, Counts of *ecTEs* and *full-length ecRepeatUnits* identified in rice across all treatment. **b,** Flower plot showing the specific and shared *ecTEs* identified among all treatments. **c,** Flower plot showing the specific and shared *full-length ecRepeatUnits* identified among all treatments. **d,** Karyoplots of the Chr08:2408800-2409200 locus showing (from top to bottom) regions of eccDNAs and Nanopore sequencing (ONT) coverage in two replicates of 3-day LN treatments (*LN_D3_1* and *LN_D3_2*), and regions of repeat unit including one *KIDDO_D* element. **e,** Karyoplots of the Chr01:25348500-25350500 locus showing (from top to bottom) regions of eccDNAs and ONT coverage in two replicates of 7-day LN treatments (*LN_D7_1* and *LN_D7_2*), and regions of repeat unit including two *KIDDO_B* elements. **f,** Karyoplots of the Chr02:29642400-29643800 locus showing (from top to bottom) regions of eccDNAs and ONT coverage in two replicates of 7-day LP treatments (*LP_D7_1* and *LP_D7_2*), and regions of repeat unit including one *KIDDO_C* element.

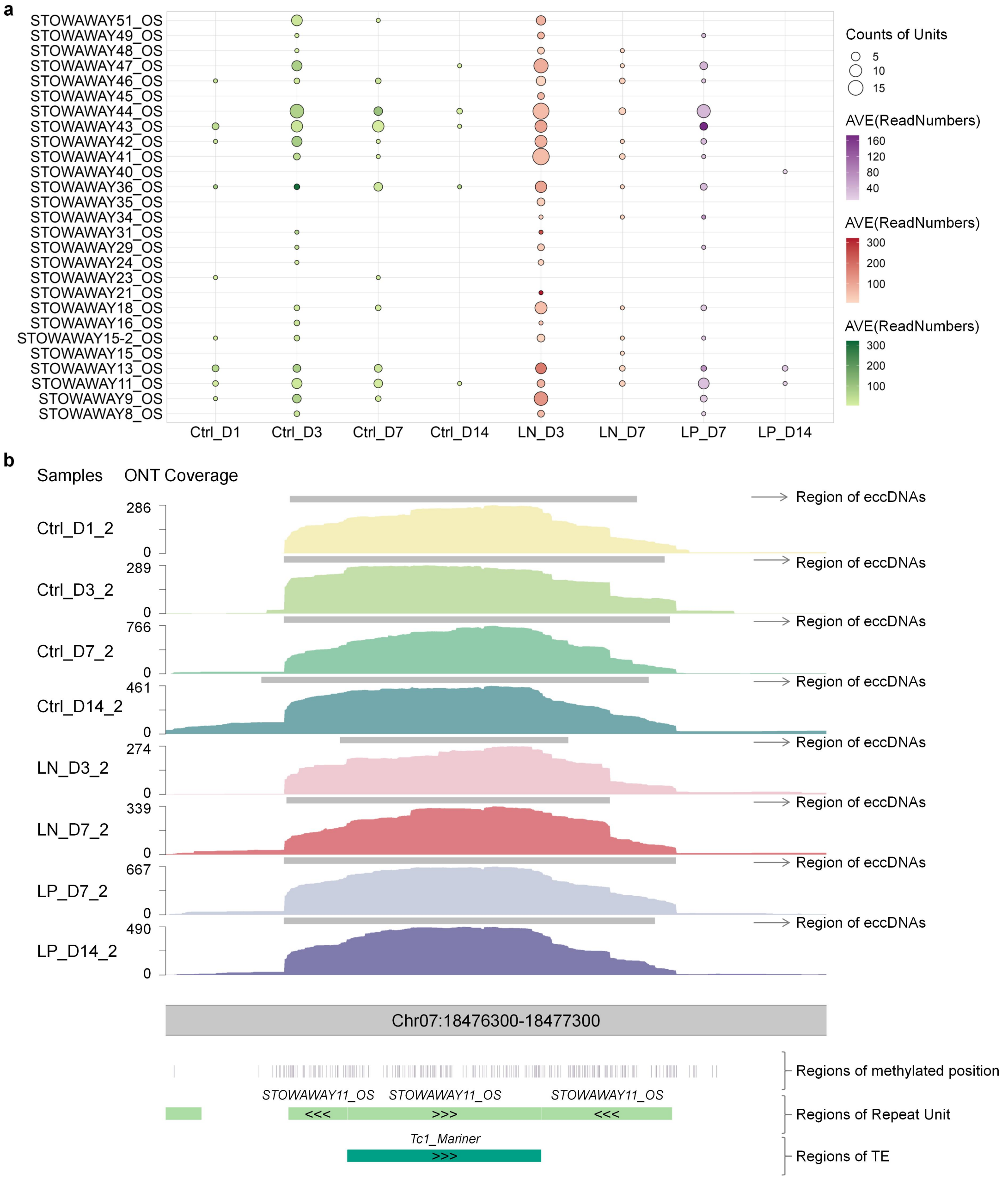

Supplementary Figure 16. Analysis of *Stowaway* in *full-length ecRepeatUnits*.
a, *Stowaway* subfamilies represented in *full-length ecRepeatUnits* across all treatments together with their repeat unit counts and read number average in two replicates of each treatment (AVE). **b,** Karyoplots of the Chr07:18476300-18477300 locus showing (from top to bottom) regions of eccDNAs and Nanopore sequencing (ONT) coverage in one replicate of all treatments (*Ctrl_D1_2*, *Ctrl_D3_2*, *Ctrl_D7_2*, *Ctrl_D14_2*, *LN_D3_2*, *LN_D7_2*, *LP_D7_2* and *LP_D14_2*), regions of methylated position, regions of repeat unit including three *STOWAWAY11_OS* elements, and regions of TE including one *Tc1_Mariner* element.

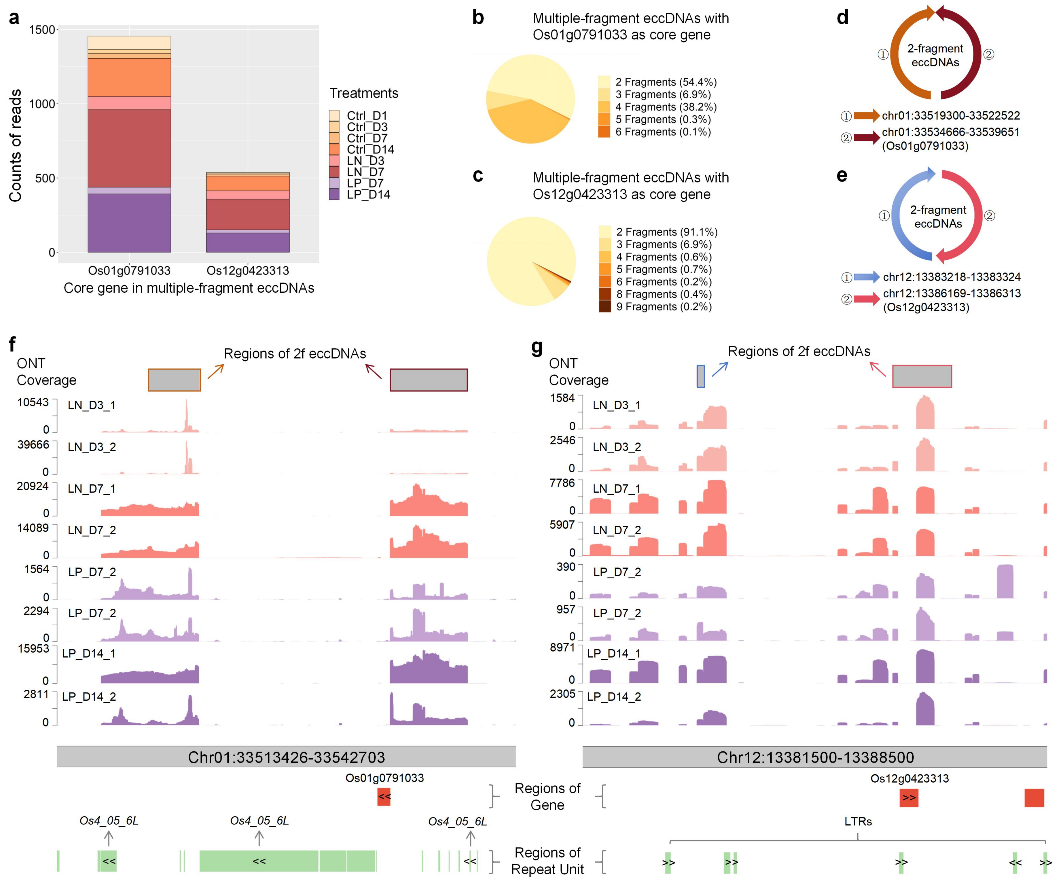

Supplementary Figure 17. Characterization and analysis of multiple-fragment eccDNAs (MF-eccDNAs).

a, Count distribution on the reads of MF-eccDNAs with Os01g0791033 or Os12g0423313 as core gene by treatments. **b,** Count distribution of the number of fragments in MF-eccDNAs with Os01g0791033 as the core gene in all samples. **c,** Count distribution of the number of fragments in multiple-fragment eccDNA with Os12g0423313 as the core gene in all samples. **d,** Circular representation and detailed fragment location of the 2-fragment (2f) eccDNAs with Os01g0791033 as the core gene. **e,** Circular representation and detailed fragment location of the 2f eccDNA with Os12g0423313 as the core gene. **f,** Karyoplots of the Chr01:33513426-33542703 locus showing (from top to bottom) regions of Os01g0791033- 2f eccDNAs, Nanopore sequencing (ONT) coverage in each two replicates of all nutritional treatments (*LN_D3_1* and *LN_D3_2*, *LN_D7_1* and *LN_D7_2*, *LP_D7_1* and *LP_D7_2*, *LP_D14_1* and *LP_D14_2*), regions of gene including Os01g0791033, and regions of repeat unit including a set of *Os4_05_6L* elements. **g,** Karyoplots of the Chr12:13381500-13388500 locus showing (from top to bottom) regions of Os12g0423313- 2f eccDNAs, ONT coverage in each two replicates of all nutritional treatments (*LN_D3_1* and *LN_D3_2*, *LN_D7_1* and *LN_D7_2*, *LP_D7_1* and *LP_D7_2*, *LP_D14_1* and *LP_D14_2*), regions of gene including Os12g0423313, and regions of repeat unit including a set of LTRs.

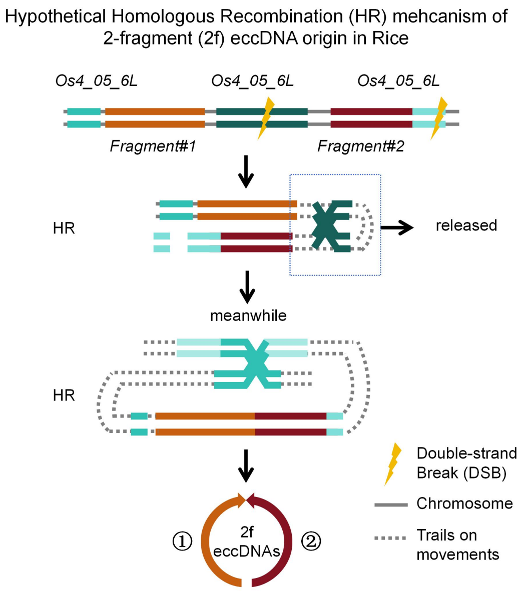

Supplementary Figure 18. Hypothetical LTR-related Homologous Recombination (HR) mechanism of 2-fragment (2f) eccDNA formation in rice.

A putative 2-fragment (2f) eccDNA origin mechanism through homologous recombination (HR) based on LTRs or their remnants in rice that similar to the mechanism previously reported for the formation of the yeast *GAP*^{circle} (lines in light cyan, cyan and deep green: three *Os4_05_6L* elements; lines and curve labeled ① in orange: one fragment in Os01g0791033- 2f eccDNAs; lines and curve labeled ② in red: the other fragment in Os01g0791033- 2f eccDNAs).
