# Supplementary material for: Adaptive dynamics of extrachromosomal circular DNA in rice under nutrient stress
Source: Nat Commun. 2025 May 4;16:4150. doi: 10.1038/s41467-025-59572-x (PMC12050283; doi:10.1038/s41467-025-59572-x)
Supplement: Supplementary file 2 — Description of Additional Supplementary Files [file 41467_2025_59572_MOESM2_ESM.pdf]

Description of Additional Supplementary Files

Supplementary Data 1. LongQC results of Nanopore sequencing

Supplementary Data 2.: Summarized GO enrichment on exclusive and differential *ecGenes* during optimal growth

Supplementary Data 3. Full-length exclusive *ecGenes* during optimal growth

Supplementary Data 4. GeneIDs shown in pathways from GO analysis on exclusive *ecGenes* [*Ctrl_D1* vs. *Ctrl_D3*]

Supplementary Data 5. GeneIDs shown in pathways from GO analysis on exclusive *ecGenes* [*Ctrl_D1* vs. *Ctrl_D7*]

Supplementary Data 6. Summarized GO enrichment on exclusive and differential *ecGenes* under LN treatments

Supplementary Data 7. Full-length exclusive *ecGenes* under LN treatments

Supplementary Data 8. GeneIDs shown in pathways from GO analysis on exclusive *ecGenes* [*Ctrl_D3* vs. *LN_D3*]

Supplementary Data 9. GeneIDs shown in pathways from GO analysis on exclusive *ecGenes* [*Ctrl_D7* vs. *LN_D7*]

Supplementary Data 10. Summarized GO enrichment on exclusive and differential *ecGenes* under LP treatments

Supplementary Data 11. Full-length exclusive *ecGenes* under LP treatments

Supplementary Data 12. GeneIDs shown in pathways from GO analysis on exclusive *ecGenes* [*Ctrl_D7* vs. *LP_D7*]

Supplementary Data 13. GeneIDs shown in pathways from GO analysis on exclusive *ecGenes* [*Ctrl_D14* vs. *LP_D14*]

Supplementary Data 14. Detailed locations on major multiple-fragment eccDNAs with Os04g0343050, Os05g0372300, Os01g0791033, Os12g0423313 as core gene

Supplementary Data 15. QC results of ATAC-seq
